# Role of Body-Fluid Biomarkers in Alzheimer’s Disease Diagnosis

**DOI:** 10.3390/diagnostics10050326

**Published:** 2020-05-20

**Authors:** Thuy Trang Nguyen, Qui Thanh Hoai Ta, Thi Kim Oanh Nguyen, Thi Thuy Dung Nguyen, Van Giau Vo

**Affiliations:** 1 Faculty of Pharmacy, Ho Chi Minh City University of Technology (HUTECH), Ho Chi Minh City 700000, Vietnam; nt.trang85@hutech.edu.vn; 2 Institute of Research and Development, Duy Tan University, Danang 550000, Vietnam; tathoaiqui@duytan.edu.vn; 3 Faculty of Food Science and Technology, Ho Chi Minh City University of Food Industry, Ho Chi Minh City 700000, Vietnam; oanhntk@hufi.edu.vn; 4 Faculty of Environmental and Food Engineering, Nguyen Tat Thanh University, Ho Chi Minh City 70000, Vietnam; 5 Department of Industrial and Environmental Engineering, Graduate School of Environment, Gachon University, 1342 Sungnam-daero, Sujung-gu, Seongnam-si, Gyeonggi-do 461-701, Korea; 6 Department of BionanoTechnology, Gachon University, 1342 Sungnam-daero, Sujung-gu, Seongnam-si, Gyeonggi-do 461-701, Korea

**Keywords:** Alzheimer’s disease, biomarkers, diagnostics, Aβ, CSF, tau

## Abstract

Alzheimer’s disease (AD) is a complex neurodegenerative disease that requires extremely specific biomarkers for its diagnosis. For current diagnostics capable of identifying AD, the development and validation of early stage biomarkers is a top research priority. Body-fluid biomarkers might closely reflect synaptic dysfunction in the brain and, thereby, could contribute to improving diagnostic accuracy and monitoring disease progression, and serve as markers for assessing the response to disease-modifying therapies at early onset. Here, we highlight current advances in the research on the capabilities of body-fluid biomarkers and their role in AD pathology. Then, we describe and discuss current applications of the potential biomarkers in clinical diagnostics in AD.

## 1. Introduction

Alzheimer’s disease is an irreversible, progressive brain disorder, characterized by the accumulation of β-amyloid (Aβ) plaques and tangles consisting of hyperphosphorylated tau protein in the brain. It accounts for 60–70% of dementia cases, making it the most common cause of dementia [1,2]. Globally, it is predicted to go up to over 131 million by 2050 [3] due to the increasing of the number of elderly. Approximately >5% of all patients with AD are diagnosed before age 65 and are grouped as early onset AD (EOAD) [4,5], while patients with AD after age 65 are grouped as late onset AD (LOAD) [4,5]. For the diagnosis of AD, these morphological abnormalities in brain cells and tissues are used for clinical evaluations. Clinical AD is mainly characterized by two key factors including intracellular neurofibrillary tangles (NFT) and extracellular amyloid plaques or senile plaques [6], with the hyperphosphorylation of tau oligomer protein [7]. In addition, amyloid plaques are suggested to be critical features of AD neuropathology [8]. With a 40–42 bp length, Aβ peptides are suggested to be the main components of these plaques [9], which result in abnormal folding of the Aβ peptides due to oligomers or fibrils in the brain tissue. In addition, in rare cases, the disease is inherited in an autosomal dominant early onset form caused by mutations in the amyloid precursor protein (*APP*), presenilin 1 (*PSEN1*), and presenilin 2 (*PSEN2*) genes, which might result in the alteration of Aβ production, leading to the apoptosis of the neurons and dementia [4,5].

Although there is no effective treatment capable of slowing down disease progression, it is believed to have a long preclinical phase, during which the affected individual has no or very subtle decline in cognition but manifests AD biomarker positivity. The characterization of the preclinical stage of AD first became possible following neuroimaging with magnetic resonance imaging (MRI) and positron emission tomography (PET), reflecting cerebral amyloidosis, tau phosphorylation and neurodegeneration. The time span of preclinical AD has, as yet, not been fully elucidated, as it seems to manifest some individual variability and could be affected by factors such as cognitive reserve, genetic profile and lifestyle. However, mounting evidence suggests that the preclinical stage is generally long, stretching over years or even decades. To alter the disease state, the drugs need to be directed towards the specific targets, and without the molecular understanding of the mechanisms and biomarkers, that is not possible [10]. Since there is no specific signature biomarker, the diagnosis of AD used to combine a multi-step process of confirming a wide range of biomarkers. The most targeted fluid biomarkers could be considered to be constituted of cerebrospinal fluid (CSF) biomarkers. However, collecting the CSF samples and confirming the biomarkers is time consuming and expensive. Thus, there should be alternative biomarkers to be used in a large number of populations. Thus, blood-based biomarkers would be the first step of the multiple steps. These biomarkers can be applied to predict the disease, and then the CSF biomarkers can contribute towards confirming the disease as early as possible [11,12]. Hence, the biomarkers become the most important factors in the early diagnosis of the disease. The purpose of this review was to provide an overview of recent body-fluid biomarkers and their role in AD pathology. Additionally, we describe current applications of the biomarkers in the development of approaches for the early diagnosis of AD.

## 2. Biomarkers in Alzheimer’s Disease

Biomarkers that can be used in vivo are essential in mapping the chain of events in AD, from the preclinical stage to the clinical stage. The characterization of preclinical AD would not be possible without the use of biomarkers, as no clinical symptoms have yet emerged. Biomarkers are also useful in selecting patients for clinical trials and for monitoring treatment effects. In the NIA-AA guidelines for AD diagnosis, the clinical use of biomarkers is optional, but it has still become widespread in the clinical setting, mainly as a supportive rather than decisive tool. This includes the CSF biomarkers Aβ42, total tau-protein (t-tau) and phosphorylated tau protein (p-tau), as well as FDG-PET, amyloid PET and structural MRI. These biomarkers reflect two of the hallmarks of AD, cerebral amyloidosis (CSF Aβ42 and amyloid PET) and neurodegeneration (CSF t-tau and p-tau, FDG-PET and structural MRI). Experimental biomarkers reflecting other pathological processes are under development, both as possible diagnostic aids and for the further characterization of the natural history of AD. These include novel CSF biomarkers and PET ligands as well as blood based biomarkers [11] and electroencephalographic algorithms [13,14]. When these Aβ plaques aggregate and cannot get metabolized they induce another cascade of inflammation [15]. In addition, urine and ocular inflammatory biomarkers in the retina of the eye may be additional biomarkers for early AD detection. Combining all the biomarkers of AD, they can be divided into a few classes like CSF biomarkers, imaging biomarkers, blood biomarkers, ocular biomarkers and urine biomarkers.

The most promising biomarkers of AD are cerebrospinal fluid Aβ peptides in conjunction with CSF tau-proteins. In AD patients, the amount of CSF Aβ protein decreases gradually with increased τ-proteins. APP has a major role in depositing amyloid plaques, and here, amyloid proteins are the metabolic products of the neurons, which are produced through cascades of cleavage reactions where the major role players are the α, β and γ secretases [16]. As previously mentioned, both Aβ and phosphorylated tau could be the main components of extracellular plaques and neurofibrillary tangles that may constitute the core biomarkers for AD detection [17]. In AD patients, the abnormal of t-tau/p-tau in the CSF may result in increased tau secretion and phosphorylation [17,18]. While the difference between topographic biomarkers (PET-FDG; MRI) and pathophysiological biomarkers (CSF, amyloid PET) have been well established [19], CSF biomarkers could be a specific physiopathological process of the disease. The presence of amyloid beta oligomer (AβO) has been correlated with synaptic plasticity impairment and frank synapse loss in mice and cell models [20,21,22,23] and in human brains in AD [24,25]. The cognitive impairment in AD closely parallels the loss of synapses due to the toxic effects of Aβ, tau, and inflammation; thus, emerging biomarkers are able to measure synapse injury and loss in the brain and may correlate with cognitive function in AD [26].

## 3. Biomarkers in the Cerebrospinal Fluid

### 3.1. Cerebrospinal Fluid Aβ

**Figure 1:**
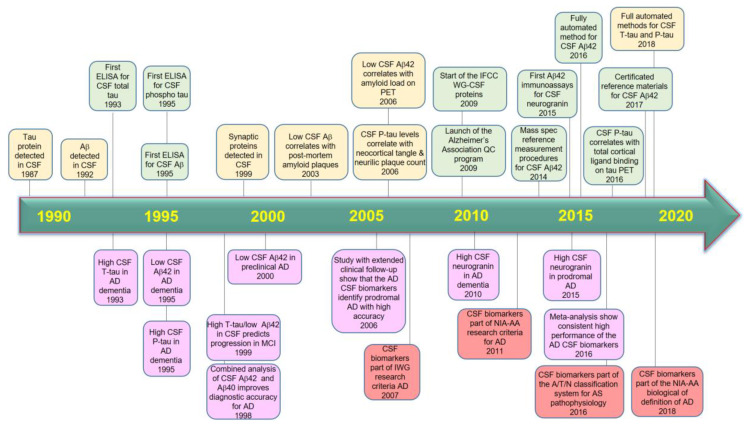
A timeline reflecting the evolution of the body fluid Alzheimer’s disease biomarkers. Green boxes, technical developments; yellow boxes, pathophysiological findings; purple boxes, clinical findings; brown boxes, evolution of clinical diagnostic criteria and pathogenic classifications. Aβ, amyloid beta; AD, Alzheimer’s disease; CSF, cerebrospinal fluid; MCI, mild cognitive impairment; N, neurodegeneration.

CSF Aβ42 may remain the quintessential fluid AD biomarkers for now, but a growing cadre of other markers are also proving their worth [27]. Three core CSF biomarkers, Aβ42, p-tau and t-tau, have been extensively evaluated for use in AD diagnosis and research. Clinically, studies have revealed that the level of Aβ42 is lower, while the figures of t-tau and p-tau are usually higher in AD patients in comparison to in healthy elderly people [28]. In addition, previous studies have reported that CSF Aβ42 decreases early in the pathological cascade of AD and remains stable and low thereafter [29,30,31,32], making it an unsuitable marker of disease severity and the rate of progression. CSF Aβ42 has been observed to already be reduced in prodromal AD and even in the preclinical asymptomatic stage of AD [33,34,35,36,37,38]. Recently, studies of biomarker classification proposed a new research framework in an effort to nudge the field toward a biological definition of AD. In 2014, the International Working Group 2 (IWG-2) for New Research Criteria for the Diagnosis of AD suggested a new conceptual framework for the presence of biomarker evidence consistent with and supportive of AD [39]. This study enabled AD diagnosis to be extended into the prodromal stage, where the disease can be diagnosed with supportive biomarkers. It adding the value of the combinations of CSF markers including t-tau, Aβ1–42 and p-tau [40] that been confirmed in large multi-center studies—namely, the Alzheimer’s Disease Neuroimaging Initiative (ADNI) study [41] and the Development of Screening Guidelines and Criteria for Predementia Alzheimer’s Disease (DESCRIPA) study [42]. The framework defined AD as the presence of plaque and tangle pathology, regardless of symptoms, and offered a systematic definition of pathological changes based on biomarkers for brain amyloid, tau and neurodegeneration. They classified them according to the A/T/N scheme into those of β-amyloid deposition, using CSF Aβ1–42 for brain amyloid (A), CSF p-tau181 for aggregated tau (T), and FDG-PET for neurodegeneration (N). Each participant’s values were deemed normal or abnormal based on the cutoffs determined in the ADNI. The binary classification of each of these three biomarker types bins people into eight possible profiles, ranging from completely negative (A−/T−/N−) to triply positive (A+/T+/N+) [10,43]. In other words, a person’s biomarker evaluation should include amyloid and tau status, and markers for neurodegeneration. How the “N” is measured will depend on local circumstances, and it is used to predict and monitor the course of the disease [44]. AD and other dementias could be distinguished based on the CSF Aβ42/Aβ40 ratio, which may improve the detection of amyloid deposition into the CSF [45,46]. However, despite Aβ maintaining its centrality in AD pathogenesis, it is not sufficient as predictor for AD [47]. Combination with other biomarkers such as t-tau and p-tau could be useful to diagnose AD cognitive decline as well as to differentiate AD from other forms of dementia more precisely. Figure 1 summarizes the evolution of the core cerebrospinal fluid AD biomarkers to date.

### 3.2. Cerebrospinal Fluid P-Tau

P-tau is the hyperphosphorylated form of the microtubule associated protein tau. The second AD CSF biomarker is p-tau, which reflects tau phosphorylation and increases by around 200% from control levels in AD, most likely before the onset of the prodromal stage, and remains steady and high thereafter [48,49]. Assays are available both for p-tau181 and at threonine 231 (p-tau231). These are considered to be equivalent in diagnostic accuracy [50], although there is a possibility that p-tau231 has a somewhat greater specificity for AD than p-tau181 [51]. The increase in CSF p-tau has, as of yet, not been observed with certainty in other pathological conditions, making elevated p-tau seemingly exclusive to AD. Interestingly, the measurement of the CSF tau/Aβ42 [52] or p-tau/Aβ42 [53] ratios could raise diagnostic accuracy and may, therefore, be suitable for routine clinical use.

### 3.3. Cerebrospinal Fluid T-Tau

The next core CSF biomarker is t-tau, which represents axonal degeneration and increases by around 300% early in the course AD, probably around the same time as p-tau [54,55]. T-tau is the least specific for AD of the three core CSF biomarkers, with increases in t-tau being observed in other neurological conditions [56]. Combining all three core CSF biomarkers yields a higher sensitivity and specificity for AD than can be achieved using only one or two of them. The combination of the reduced concentration of Aβ42 and high concentrations of p-tau and t-tau comprises the so called “AD signature” in CSF, which has been shown to be highly predictive of progression to AD dementia in mild cognitive impairment (MCI) patients [41,54,57,58,59]. An example of this is a study showing a 95% sensitivity and 87% specificity of the “AD signature” in the CSF in distinguishing between prodromal AD and stable MCI [59]. Some CSF candidate biomarkers for AD diagnosis are summarized in Table 1. 

## 4. Biomarkers in Blood

### 4.1. Plasma Aβ

CSF biomarkers have dominated the biomarkers, with low levels of CSF Aβ42 showing a reliable correlation with AD risk [64]. Amyloid in the blood, on the other hand, has yielded a more confusing picture. Some studies have found a relationship between low levels of plasma Aβ and the development of Alzheimer’s [65,66,67,68,69,70], including one from last year that followed more than 1000 elderly people in France over four years [71]. Other work, meanwhile, has shown the opposite pattern, with high levels of plasma Aβ correlating with disease risk [69,72,73,74]. One possible explanation that has been put forth for this discrepancy is that plasma Aβ might be high initially in people at risk for AD [69,72]. Several studies have failed to find any strong association between plasma Aβ and cognition [68,69,70,72,73,74].

**Figure 2:**
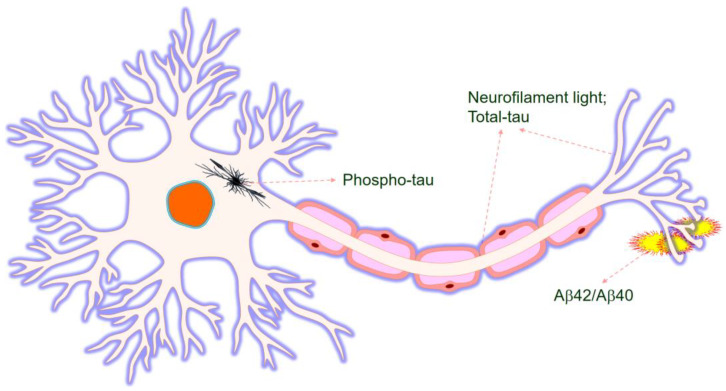
Neurofilament, tau and Aβ aggregation as indicators in the AD brain.

Currently, AD is typically detected and diagnosed through memory and cognitive tests or MRI and PET scans of the brain, which can show evidence of tau or amyloid buildup. As mentioned above, although CSF biomarkers provide a more accurate diagnosis of AD, blood biomarkers have advantages over the CSF biomarkers [15,18]. In addition, blood samples can be very easily collected from the patients, while the collection of the CSF samples needs procedures that require lumber punctures. Therefore, it becomes a great drawback of the CSF biomarkers in comparison to the blood biomarkers [75,76] due to its stability. However, assaying Aβ proteins in the blood becomes difficult when other proteins present in the blood interfere with the procedure, or the sample can even undergo photolytic reactions [18]. Figure 2 summarizes AD biomarkers in the blood.

A range of blood-based Aβ assays have been recently developed based on immunoassays, promising for early AD detection [77]. To distinguish AD samples and controls through the Aβ42/Aβ40 ratio, several assays have been developed and applied to detect Aβ40 in the plasma such as a single molecule array (Simoa) [78,79], immunomagnetic reduction (IMR) [80,81] or enzyme-linked sandwich immunoassay (ELISA) [82]. An additional plasma Aβ test, an immunoprecipitation method, was developed and able to measure abnormal total Aβ in the plasma [83]. Using those techniques could measure Aβ aggregates or Aβ bound to other proteins at the low amounts in the AD plasma [80,84].

### 4.2. Plasma Tau

Many studies have reported that phosphorylated tau turns up in peoples’ plasma decades before they show signs of dementia. The data bore the promise of a blood test for early Alzheimer’s disease—one that is simpler than measuring the notoriously finicky plasma Aβ, and more specific than neurofilament light [27,28,34,59,60,85,86,87,88]. In AD patients, plasma tau concentrations are increased compared with in normal controls, which can be measured using ultrasensitive assays but not as clearly as in the CSF [89]. The relationship of plasma T-tau with disease in 458 participant patients was measured by the Simoa approach [90], which may associate with incident AD [90]. The p-tau concentration was associated with both Aβ and tau PET in AD patients, and it was possible to detect tau phosphorylated at amino acid 181 by an assay [91].

Plasma p-tau181 was associated with CSF p-tau181 and with tau PET, and it predicted further cognitive decline among people who were already mildly impaired. In this study, plasma p-tau181 was much more specific than plasma neurofilament light, a promising marker for neurodegeneration. People with AD had more p-tau181 in their blood than did non-diseased controls, and it correlated with their PET measures of amyloid plaques and neurofibrillary tangles [91]. In fact, plasma p-tau181 better correlated with amyloid than did the plasma Aβ42/Aβ40 ratio, indicating that this particular phospho form of tau reflects ongoing Aβ pathology. Recently, a study reported that p-tau181 and p-tau217 rise in the CSF of autosomal-dominant AD cases 19 and 21 years before symptom onset, respectively [92]. Plasma p-tau181 distinguished AD from all other diseases with a sensitivity and specificity of 92 and 87 percent, respectively, and an area under the curve of 0.92. These findings show that plasma p-tau181 increases early in AD, around the timepoint of Aβ positivity, and support plasma p-tau181 as a possible early marker of AD [93,94,95].

### 4.3. Other Biomarkers in Plasma

Given that Aβ42 and tau are specific markers of AD pathogenesis, the utility of a marker of neuronal death in the diagnosis of AD was suggested recently [96]. Besides the core AD biomarkers CSF Aβ42, Aβ40, p-tau181 and total tau, the markers include α-synuclein as a gauge of synaptic dysfunction; S100b, YKL-40 and glial fibrillary acidic protein (GFAP) [97] as markers of astrocyte activation; soluble TREM2 and IL-6 as markers of microglial activation and inflammation [98]; and neurofilament light (NfL) and neurogranin [99] as markers of axonal injury and synaptic dysfunction. Recently, specific protein biomarkers of synapse degeneration such as SNAP-25 and synaptotagmin in the CSF have been developed [23,100,101]. Other synaptic proteins including SNAP25, RAB3A, GAP43, AMPA receptor subunits, and a number of other proteins also show promise as CSF biomarkers of synaptic damage and loss [102,103,104,105]. The investigators vote new candidate biomarkers into this jointly evaluated set as individual research labs produce promising evidence for them. In addition, plasma clusterin, which is also called Apolipoprotein-J (APOE-J) [106,107], is mainly associated with the clearing of debris from the brain [108]. When the clusterin gene is modified or mutated, it becomes a risk factor for AD [106]. However, on which allele the mutation occurrs is still unclear. Clusterin exerts a protective function on the neurons, and it was suggested that it modifies the amyloid-β aggregates by clearing them away [106,107]. It was also found that high clusterin levels exert protective functions in younger persons whereas they exert toxic functions in older patients. Therefore, there is an increasing burden of AD pathogenesis when the amount of CSF clusterin is increased for exerting protective functions and inhibiting apoptosis [109]. In response to the increased CSF clusterin, there is also an increased amount of plasma clusterin [110]. As a result, with the development of the severity of the disease, there is an increasing amount of plasma clusterin [110]. However, it is still not completely determined if plasma clusterin can diagnose AD at the initial level [111]. Rather, it has been suggested that the clusterin starts to increase in amount when the disease has already progressed. Thus, it can be used as a prognostic biomarker rather than a diagnostic biomarker [107].

## 5. Other Potential Biomarker Sources

Along with affecting the brain mostly, AD also affects the eyes. The retinas of the eyes contain neurons that extend to the brain and render visual signals to the brain. As a result, the proteins responsible for AD pathology are also present in the retina. Therefore, the eye becomes easier to access than the brain imaging. Moreover, it a non-invasive process, which means the ocular biomarkers may be useful for the diagnosis of the AD [112,113]. Several potential ocular biomarkers could be assessed for the diagnosis of the AD, such as retinal Aβ accumulation, the loss of the retinal ganglion and the nerve fiber layer, retinal vascular biomarkers [114,115,116,117,118] and lens biomarkers [119,120,121].

Previous studies revealed that Aβ plaques could be found in the retina in both animal models, and in humans, the retinal dysfunction was caused in AD by the accumulation of amyloid-β proteins in the retina, which alters the normal structural functionality of the retina [122]. In almost all layers of the retina, the aggregation of Aβ plaques was identified. In another study, a South American rodent showed brain Aβ, tau-accumulation, and cognitive impairment due to age and also retinal amyloid-β accumulation and tau accumulation, more specifically in the nerve fibers and ganglionic cells. Moreover, in other animal studies, the Aβ plaques in the case of AD appeared in the retina even before appearing in the brain. In some studies, it was possible to confirm AD using the Aβ accumulation in the retina, though the number of the studies is very small. More extensive studies and confirmations are needed to establish retinal Aβ deposition as an invasive biomarker for the detection of AD [123]. In addition to the clinically most relevant biomarkers, t-tau, p-tau and Aβ42 are discussed, as is how they may be used, together with other diagnostic investigations, to make a pre-dementia diagnosis of and screening test for AD early on.

## 6. Conclusions and Future Perspective

As there is no cure for the disease, the prevention of the progression of and diagnosis of AD at the earliest stage is very crucial. While the exact cause of the disease, risk factors and molecular understanding of the disease are still very unclear, discovering the biomarkers very specific to the disease is very important, and for diagnosing such a complex disease, it is not possible to depend on only one form of biomarker. An attractive next step in studying biomarker changes in preclinical AD would be to embark on a larger multi-center longitudinal study. The subjects would then be followed up at previously defined intervals with the repeated collection of CSF and blood and with neuroimaging and neuropsychological assessments. Through such prospective collection of data from a large number of individuals, where a substantial part of the study group is expected to develop clinical AD during follow-up, one would be able to map the temporal trajectories of preclinical biomarker changes with precision, thereby gaining valuable knowledge on the true course of events during the development of AD.

The representative biomarkers of AD are Aβ42, Aβ40, total tau protein and phosphorylated tau protein, and the concentrations of these biomarkers in human plasma are directly correlated with the pathology of AD. As the disease is mainly neural related, the CSF biomarkers are the closest ones to the disease site. The major CSF biomarkers of the disease are the Aβ aggregates and tau protein deposition. The Aβ aggregates can also be found in other types of dementia as well, but the amount might vary, whereas tau protein deposition is very specific towards AD and is very useful in differentiating AD from the rest of the kinds of dementia. However, collecting these biomarkers is not easy; it is both time consuming and expert-dependent. Once these biomarkers are accurately correlated with the disease, they will be the easiest accessible biomarkers for the diagnosis of AD. Therefore, new biomarkers are needed to track non-Aβ and non-tau pathologies. Many proteins involved in the pathophysiological progression of AD have shown promise as new biomarkers. Circulatory microRNAs [124], ceramides [125], neurogranin [126], neuronal pentraxin 1 [127], serum beta-secretase 1 [128], neuronal pentraxin 2 [127], APP 669–711/Aβ 1–42 [129], APOE4 [130], albumin ratio [131], α-synuclein [132], proteostasis-related biomarkers [133,134], and visinin-like protein 1 [135,136] could aid the prediction of AD progress as well as early diagnosis (Table 2). These have direct connections to the CNS; therefore, accessing the ocular biomarkers will be helpful to easily access the CNS biomarkers.

By measuring the concentrations of AD biomarkers simultaneously, Alzheimer’s patients can be discriminated from health controls with a high sensitivity, selectivity and accuracy; there are available techniques that have been developed and approved for clinical diagnostic and screening studies. Another interesting future direction would be to collect and characterize more cases of suspected reduced penetrance of familial AD mutations found across the globe. With a larger number of reduced penetrance cases, it would be possible to pursue the underlying factor causing these individuals to be spared from developing EOAD.

Although there are many biomarkers that are expected to correlate with AD pathology, those are still being investigated as to their exact relation with the AD, which is still not clear. On the other hand, suspected non-Alzheimer’s pathophysiology (SNAP) has interested the field, since patients have one of several markers of neurodegeneration but test negative for brain amyloid and have not been diagnosed with a specific neurodegenerative disorder [137]. Based on current data, it appears that cognition in people with SNAP deteriorates much more slowly, if at all, than in those who have both brain amyloid and neurodegeneration [138]. Thus, there should be further confirmatory studies to establish the biomarkers and to make the diagnostic procedures more efficient. Hence, there should be more emphasis on discovering the correlations between the disease and non-invasive biomarkers and blood biomarkers, as in many of the countries, the imaging biomarkers and CSF biomarkers are still not easily accessible and are costly and, most importantly, very time consuming. In this situation, with the annually increased number of Alzheimer’s patients, establishing non-invasive and circulatory biomarkers will improve the situation towards the betterment of AD. Combining biological markers and neuro-imaging improves diagnostic accuracy and reduces the number of individuals needed for a clinical trial of a new treatment.

## Figures and Tables

**Table 1:** The presence of Aβ42, p-tau and t-tau in CSF corresponds with pathology as core biomarkers (Notes: ↑, increased; ↓, decreased).

Sample Size	Biomarker(s)	Cut-Off	Sensitivity	Specificity	Reference
21 AD	↓ Aβ42	Aβ42: < 427 ng/L	86%	88%	[60]
	↑ t-tau	t-tau: < 445 ng/L	86%	88%	
24 normals	↑ p-tau	p-tau: < 74 ng/L	60%	88%	
180 MCI	↓ Aβ42 ↓ p-tau181	t-tau: > 50 ng/L	95%	83%	[59]
57 MCI-AD	↑ t-tau	p-tau181: > 60 ng/L			
137 MCI	↓ Aβ42	Aβ42: ≤ 0.64 ng/mL	93%	53%	[59]
	↓ Aβ42/Aβ40 ratio	Aβ42–Aβ40: ≤ 0.95	87%	78%	
529 AD	↓ Aβ42	Aβ42: ≤ 482 ng/l	79%	65%	[61]
304 normal	↑ t-tau	t-tau: ≥ 320 ng/l	84%	47%	
271 AD	↑ p-tau	p-tau: ≥ 52 ng/l	86%	56%	
24 AD	Aβ42	t-tau: > 325.7 pg/mL	83%	91%	[62]
76 AD	↑ t-tau	Aβ42: < 481 pg/mL	94%	87%	[63]
47 dementia	↑ p-tau	t-tau: > 326 pg/mL	84%	96%	
		p-tau: > 57 pg/mL	72%	90%	
		t-tau/Aβ42: > 0.55	99%	95%	
		p-tau/Aβ42: > 0.10	96%	96%	
		t-tau/Aβ42: > 0.08l	93%	70%	

**Table 2:** Summary of the current development of body-fluid biomarkers for Alzheimer’s Disease.

Biomarker	Measuring	Method for Measurement	Monitoring	Stage of Development
Aβ40 amyloid and total Aβ42 amyloid, free, bound, free/bound, truncated, sAPPα	Blood/plasma	Amyloid	Diagnostic, prognostic, predictive	Clinical trials
Fatty acid binding protein 3	CSF	Neuronal damage	Diagnostic	Clinical trials
Circulatory microRNAs	Blood/plasma	Cell signaling	Unknown	Preclinical
Multi-parameter diagnostic blood test	Blood/plasma	Unknown	Diagnostic	Clinical trials
Ceramides	Blood/plasma	Inflammation	Diagnostic	Clinical trials
Neocortical β-amyloid burden	Blood/plasma	Amyloid	Susceptibility/risk	Clinical trials
Blood brain barrier	Blood/plasma, imaging, CSF	Vasculature	Diagnostic, monitoring	Clinical trials
Blood biomarker for mtDNA Damage	Blood/plasma	Genetic variation/DNA	Diagnostic, monitoring	Clinical trials
Neurogranin	CSF	Neuronal damage	Diagnostic, susceptibility/risk	Clinical trials
Neuronal pentraxin 1	Blood/plasma	Neuronal damage	Diagnostic	Clinical trials
BACE1	Blood/plasma	Amyloid	Diagnostic, susceptibility/risk	Clinical trials
Neuronal pentraxin 2	CSF	Inflammation	Diagnostic	Clinical trials
APP 669–711/Aβ 1–42	Blood/plasma	Amyloid	Prognostic	Preclinical
APOE4	Blood/plasma, other bodily fluids	Genetic variation/DNA	Susceptibility/risk	In use (FDA approved)
Neuron specific enolase	CSF	Neuronal damage	Susceptibility/risk, predictive	Clinical trials
Albumin ratio	Blood/plasma, CSF	Amyloid	Diagnostic	Clinical trials
Aβ42/Aβ40 (Plasma)	Blood/plasma	Amyloid	Pharmacodynamic/response, susceptibility/risk, safety	Clinical trials
Aβ1–42/Aβ1–40 (CSF)	CSF	Amyloid	Diagnostic, prognostic	Clinical trials
Aβ42 (salivary)	Other bodily fluids	Amyloid	Diagnostic, prognostic	Clinical trials
Aβ42 (blood)	Blood/plasma	Amyloid	Diagnostic	Clinical trials
Aβ42 (CSF)	CSF	Amyloid	Diagnostic, prognostic, susceptibility/risk	Clinical trials
Aβ1–17 (Aβ17)	Blood/plasma	Amyloid	Diagnostic	Preclinical
Plasma lipoproteome	Blood/plasma	Neuronal damage	Diagnostic	Clinical trials
α-synuclein	CSF	Amyloid	Diagnostic	Clinical trials
Proteostasis-related biomarkers	CSF	Amyloid, inflammation, neuronal damage, Tau	Diagnostic, monitoring, susceptibility/risk	Clinical trials
Tau in the biological fluids	Blood/plasma, CSF	Tau	Diagnostic, monitoring	Clinical trials
TNF-α (plasma)	Blood/plasma	Inflammation	Diagnostic	Clinical trials
Vascular cell adhesion molecule 1	Blood/plasma	Neuronal damage	Diagnostic, monitoring, prognostic	Clinical trials
Visinin-like protein 1	CSF	Neuronal damage	Pharmacodynamic/response	Clinical trials
